# “Are We to Treat Human Nature as the Early Victorian Lady Treated Telegrams?” British and German Sexual Science, Investigations of Nature and the Fight Against Censorship, c. 1890-1940^1^

**DOI:** 10.7560/JHS33105

**Published:** 2024-01

**Authors:** Kate Fisher, Jana Funke

**Affiliations:** University of Exeter

## Introduction

Histories of sexology often examine moments of censorship in which sexological knowledge was repressed, banned or destroyed. Familiar examples include the Bedborough trial, which resulted in the censorship of John Addington Symonds’s and Havelock Ellis’s *Sexual Inversion* (1897) in England,^3^ or the ban of the German film *Anders als die Andern* (1919), co-written by and starring Magnus Hirschfeld.^4^ The violent destruction of Hirschfeld’s Berlin Institute of Sexology by the Nazis in 1933 has come to be seen as a defining moment of erasure not only within histories of sexology, but queer and trans histories more widely.^5^ To be sure, concerns about publishing and disseminating sexological research and the dangers of censorship are everywhere in the archival records, and these famous cases are not isolated incidents. There can be no doubt that the threat and reality of censorship restricted the production and circulation of sexological knowledge in fundamental ways.^6^ At the same time, as we argue in this article, scientists and others producing and circulating sex research in early twentieth-century Britain and Germany were not uniformly opposed to the censorship of sexual knowledge. On the contrary, many sex researchers conceded that who had access to sexual knowledge, where and when needed careful regulation. This view was informed by an understanding of human nature and the sexual instinct as changeable and open to influence. Indeed, the foundational idea of sexology – that scientific knowledge of human sexuality was of vital importance, and that sexual science should guide the organization of sexual life – hinged on the assumption that human nature and the sexual instinct needed to be governed and controlled through scientific knowledge.

Building on Foucault’s work, scholars investigating the circulation of sexual knowledge have long insisted that censorship can never be understood as a singular repressive force that fully succeeds in eradicating ideas without engendering forms of resistance.^7^ The enabling and constitutive functions of censorship have been widely discussed in relation to nineteenth- and early twentieth-century culture.^8^ Our article builds on existing scholarship by demonstrating that censorship regimes inspired early twentieth-century sexual science to challenge definitions and boundaries of the indecent, obscene or pornographic.^9^ The threat of censorship compelled sexual scientific writers to ask questions about the influence their work may have on readers and to develop innovative strategies of representing, publishing and circulating sexual knowledge. In this regard, censorship needs to be recognized as a constitutive factor driving the emergence of sexology. The very nature of sexual science, its driving questions, institutional structures and research methodologies were developed in the context of and in dialogue with the constraints and opportunities offered by the politics of censorship. At the same time, sexual scientists did not merely respond to these communication cultures, but often agreed with the underpinning premises that governed censorship practices and actively contributed to the politics of censorship. Indeed, for many sexual scientists, the findings of sex research provided evidential support for claim that sexual knowledge required careful regulation. We argue that sex researchers’ views on censorship were shaped, in particular, by models of the sexual instinct that underpinned their work and by the role that constructions of “nature” and the “natural” played in this understanding.^10^

As we will go on to demonstrate, appeals to nature and the natural featured frequently in decision-making about whether or not sexological ideas should be published and circulated among different audiences.^11^ First, presenting sexual desire and intimacy as natural elements of human life supported sex researchers’ claim that sex was an appropriate and necessary object of scientific study. Second, the assertion that sex was natural was used to counter and upend the argument that sexological knowledge was potentially dangerous or damaging. Sex researchers suggested that those who sought to protect individuals from sexological findings were preventing people from having access to vital knowledge that could improve individual and social health and morality. According to this logic, it was the censors who, unable to appreciate the natural truth of sex, imbued it with “seedy” and “unclean” associations. This robust defense of sexology as a beneficial science of human nature, however, also led to intellectual incoherencies that meant that sex researchers often remained complicit with arguments in support of censorship. In attacking repressive approaches to sexual knowledge as damaging, they accepted and reinforced the premise of many arguments in favour of censorship, namely, that natural sexual impulses needed guiding, since the sexual instinct was a volatile and changeable force that could easily be influenced and even corrupted by external influences. The recognition that sexual knowledge held the potential to shape the sexual instinct resonated with intense sexological debates about whether, and to what extent, sexual desire was inborn or acquired. Acknowledging this construction of sexual desire meant conceding that there were circumstances and contexts within which sexological knowledge itself needed to be regulated to prevent the sexual instinct from being misdirected. As a result, sex researchers – as well as their publishers, reviewers and readers – agreed with and, at times, actively developed censorship strategies to control the production and circulation of sexological knowledge. This was not simply because sex researchers caved in to external pressures, but because the very understanding of the sexual instinct as open to influence was key to the legitimation of sexual science. The idea that human sexuality could be shaped and altered meant that it required careful scientific guidance, which sex researchers promised to provide.

## Sexual Knowledge, Instinct and Corruption

Although there were significant national differences in the legal frameworks governing the publication of sexual scientific works across Europe and North America, one key concern underpinning justifications of censorship were anxieties about the corruption or seduction of populations perceived as vulnerable to influence, especially children and young people, women and working-class men.^12^ In Britain, the 1857 Obscene Publications Act together with the 1868 Hicklin Test established that all materials tending “to deprave and corrupt those whose minds are open to such immoral influences” were obscene – irrespective of a text’s overall merit or the author’s intent. Late eighteenth-century censorship protocols in German states, including Prussia and Bavaria, similarly foregrounded concerns about the “corruption of morals” and about the need to protect the imaginary figure of the “innocent (and vulnerable) reader” from damaging external influences.^13^ Post-unification, Germany’s first national criminal code of 1872 and subsequent rulings associated obscenity with “injured feelings” and a tendency to inspire immoral acts.^14^

More specifically, debates about censorship of sexual information frequently centered on the question of whether obscene texts could sexually corrupt susceptible individuals and inspire people to engage in illicit sex acts.^15^ Underpinning these concerns was the assumption that obscene textual passages would compel vulnerable readers to respond instinctively and trigger processes of decay and degeneration.^16^ Given the understanding of sexual health as affecting both the individual and the species, this possibility of corruption gave rise to grave concerns. As a result, the notion of the sexual instinct as a potentially precarious and volatile force that, at least in some individuals, responded mechanistically to external stimuli meant that sexual knowledge needed to be carefully controlled and managed.

In England, obscenity laws were vaguely defined and could be wielded to prosecute a very wide range of publications. Importantly, a text could be deemed obscene irrespective of whether the publication had scientific or artistic merit and regardless of the amount of obscene content. The result was an environment in which sex researchers hoping to write openly about sexual matters had to navigate the threat of their work being labelled as obscene and censored.^17^ The law was unsystematically applied, and prosecutions were haphazard and hard to predict. For instance, radical free-thinkers Annie Besant and Charles Bradlaugh were arrested and charged for breaching the Obscene Publications Act in 1877 for publishing and distributing a new edition of the birth control pamphlet *The Fruits of Philosophy* by Charles Knowlton. The judgment was then rescinded due to a technicality in the wording of the original indictment.^18^ In contrast, as mentioned above, Symonds’ and Ellis’s *Sexual Inversion*, often considered to be the first book-length treatise on homosexuality published in England, was deemed obscene, and the publisher, George Bedborough, was convicted.^19^ In 1909, the English publishers of Eden Paul’s translation of German dermatologist Iwan Bloch’s *The Sexual Life of our Time* found themselves on trial, charged with the dissemination of a work of obscenity.^20^ The prosecution was successful and copies of the work were ordered to be seized. Having threatened to appeal, a compromise was struck: the book remained on sale, but the publishers agreed to add a new prefatory note, explaining that the work was offered to booksellers in the expectation that sales would be restricted to members of the medical profession. These negotiations with the censor created a, sometimes overstated, sense that it was impossible to publish sexual scientific texts in England. While many works of sexual science remained on sale, complaining about the struggles of censorship served sexologists’ self-presentation. After the Bedborough trial, for instance, Ellis went so far as to complain that his “own country should be almost alone in refusing to use the conditions of reasonable intellectual freedom”.^21^

The notion that England had harsher censorship laws than other European countries was not entirely untrue. In post-unification Germany, legal opinions tended to be more nuanced, considering the meaning of a text in its entirety rather than based on isolated passages and accepting the validity of publishing materials serving scientific and educational purposes.^22^ As a result, most sexological pamphlets, journals and books were deemed as suitably serious and scientific in Wilhelmine Germany.^23^ Prosecutions were more frequently brought against mass-produced works, such as photographs, postcards, films and magazines and publications aimed at non-specialist readers. There were ongoing concerns about accessibly written and publicly advertised sex advice manuals and birth control literature, which tested unstable boundaries between the scientific and the obscene.^24^ The attempted censorship of German jurist and reformer Karl Heinrich Ulrichs’ publications, which were also disseminated in the form of relatively inexpensive non-specialist pamphlets rather than in scientific journals or as high-priced scholarly books, was unremarkable. However, in this case, the prosecution seeking to censor the pamphlets failed, since the Leipzig court that heard the case deemed the works did not seek to encourage immorality.^25^ The illustrated homophile magazine *Der Eigene*, which contained sexual scientific ideas, was successfully tried for obscenity three times (1900, 1903 and 1906) on the grounds that its defenders failed to demonstrate that the material did not glorify pederasty.^26^ Even though it was undoubtedly easier to publish sexual scientific material in Germany, concerns around the corrupting influence of explicit sexual writings and uncertainties about where to draw the line between the scientific and educational and the obscene or pornographic abounded and shaped the emergence of sexual scientific networks.^27^

Although the understanding of the sexual instinct as needing careful organisation and control underpinned arguments in favour of censorship, the very same conceptualisation could also be mobilised by sex researchers to establish the urgent need for reliable and authoritative scientific knowledge about sex to be produced and circulated. If the sexual instinct was a dynamic force that responded to external stimuli, society had to ensure that it had sufficient knowledge and understanding to guide and channel the sexual instinct to protect the health of the individual and the advancement of the species. This allowed sex researchers to argue that sexual science was a vital project that required sustained and widespread dissemination. The volatile nature of the sexual instinct, and the need for it to be regulated to ensure that it could aid development and progress, provided a powerful justification for sexual scientific research. It was indeed the changeable nature of the sexual instinct that informed the characterisation of sexual science as a reformist project, which sought to harness sexual knowledge to change sexual cultures, regulatory frameworks, and educative practices.

This did not mean, however, that sex researchers were simply opposed to censorship or that they advocated uncontrolled publication of their works. Acknowledging that the sexual instinct needed to be guided and controlled meant that the circulation of sexual scientific works required careful management. Sex researchers had to toe the line between championing the dissemination of their work and recognising the need for sexual knowledge to be regulated to ensure that the appropriate messages were provided to the right people at the correct time. The conflict here was not between an imagined group of liberationist sex researchers and a separate camp of moralising anti-censorship campaigners. Instead, the sources reveal a general consensus that the dissemination of sexual scientific materials needed to be carefully discussed and potentially regulated, since sexual knowledge could have a powerful impact on the people receiving it. As a result, it was not simply opposition to censorship, but contestations around the impact and dissemination of sexual scientific knowledge that played a fundamental role in shaping the emergence of late nineteenth- and early twentieth-century sexual science.

## Sexology as the Scientific Study of Nature

Sex researchers frequently appealed to nature to position sexual science as appropriate, valuable and necessary rather than dangerous, damaging and corruptive. Sexual scientific knowledge, they asserted, simply revealed basic facts about human and animal nature. This was a key goal central to modern science, which had to explore all aspects of nature, including sexuality, even if this meant addressing unpalatable or scandalous topics. In 1890, Ellis argued in his first book *The New Spirit* that the modern world had abandoned the “curious dread of all attempts to face simply and sincerely the facts of life”, adding that “wherever science goes the purifying breath of spring has passed”.^28^ Italian neurologist and anthropologist Paolo Mantegazza justified sex research in similar terms in 1885, stating: “All that is human is the province of science. […] For science, the obscene does not exist.”^29^ When Ellis’s and Symonds’ *Sexual Inversion* was censored as obscene in the UK, various European scientists condemned the ban along these lines. French physician Charles Féré, for instance, called the text “scientific” and not “immoral” since “truth is always moral and good; in seeking truth science cannot be either immoral or bad”.^30^

These claims go beyond the justification of sexology being “objective” or “scientific” in terms of specific methods. In fact, when appealing to the modern spirit of science, sex researchers were not making a statement about the value of particular methods of study and, indeed, there was no consensus about which approaches were most suitable to study sexuality scientifically. Instead, sex researchers were making a broader statement about the value of studying all of human nature and the natural world without fear of causing offence or breaking taboos. Anything found in nature could not in principle be pornographic or obscene, since it was by definition free from artifice or corruption. German entomologist and anthropologist Ferdinand Karsch-Haack, for example, articulated this by positioning nature as the only authoritative voice able to determine what was and what was not appropriate to study: “false Christianity”, he insisted, misunderstood science, yet the true scientist is unaffected by such attacks on their propriety, since, “the natural scientist, the explorer of truth […] recognizes only one judge, nature”.^31^ This rhetoric was adopted by various types of scientific writing, including early twentieth-century eugenic contributions to debates on sexual morality. English hygienist M. E. Robinson’s “The Sex Problem” of 1911 reassured readers that when “dealt within a scientific spirit, the facts [of sexual life] arouse no more emotion than other facts in nature, and the manner of approaching them need never be ‘frank’ or ‘delicate’ or ‘skilful“’.^32^ The rhetoric of nature allowed sex researchers to maintain that any aspect of natural life needed to be included within scientific study, including seemingly taboo aspects, which would cease to be unpalatable if they could only be recognized and discussed dispassionately as part of nature.^33^

This appeal to nature was often part and parcel of an established evolutionary tradition of knowledge which championed looking at all elements of life, including seemingly taboo aspects, because anything that is part of nature needed to be part of scientific knowledge.^34^ This is evidenced by the anonymously published *Darwin on Trial at the Old Bailey*, a text written in response to the Bedborough trial and the censorship of *Sexual Inversion* in England. The book positions works of sexual science within longer traditions of “anthropological and biological research”, including work on the “science of reproduction and propagation” that, the text claims, began with Darwin.^35^ It makes the strong case that “emotions as beautiful and natural as the singing of birds and the blossoming of flowers” need to be discussed openly and without shame.^36^ German sex researchers also legitimated their work by evoking the authority of evolutionary scientists like Ernst Haeckel. Hirschfeld’s popular *Die Naturgesetze der Liebe* (1912), for instance, was dedicated to Haeckel and cites correspondence between Hirschfeld and Haeckel in which the latter stresses the importance of scientific investigations of sexual life.^37^

For many, sexual scientific knowledge had a value that necessitated its circulation beyond professional circles. Sex – as a natural element of all human existence – had to be understood by the wider population and not just medics, psychiatrists or jurists. Many sexological networks and publications emerged specifically to share sexological knowledge with select publics. The British Society for the Study of Sex Psychology (BSSSP), for instance, was partly founded to provide a lecture series and lending library to adult members eager to learn more about the latest sexological research.^38^ Much of the correspondence reveals the efforts undertaken by members of the BSSSP to publish and circulate sexological publications nationally and internationally, often in the face of censorship. The BSSSP corresponded with the British Museum to ensure that members could have access to the private case in which sexological and other allegedly obscene publications were stored. This had already been noted as a problem by English poet and socialist reformer Edward Carpenter, who discovered in 1912 that his book *The Intermediate Sex*, which he had donated to the British Museum, was not available to readers, since it had been locked away in the private case. He engaged in a lengthy correspondence with the British Museum about the material that was excluded from their public catalogue.^39^ This correspondence formed the basis of a polemic article Carpenter wrote for the *English Review*. His argument did not concentrate on the value of his book for medical authorities – although he noted how well received it had been by them – but rather the interest from teachers and other educationalists.^40^ For Carpenter, sexual knowledge, even and especially of homosexuality, was essential knowledge about nature that needed to be part of the curriculum, read and disseminated by all educators.

In the later 1910s and 1920s, English and German sex researchers continued to try and reach broader audiences by writing in a more accessible style and by using new media like the cinema to disseminate their findings more widely, which often resulted in particular pushback from censors.^41^ Literature also served as a vehicle for disseminating sexological ideas.^42^ Radclyffe Hall’s *The Well of Loneliness* (1928), for instance, was published with a brief prefatory ‘Commentary’ by Ellis, and the novel’s protagonist, Stephen Gordon, identifies as a ‘sexual invert’. James Douglas, editor of the *Sunday Express*, successfully called for the book to be censored in Britain, arguing that the circulation of sexological knowledge needed to be restricted to scientific textbooks in order “to prevent the contamination and corruption of English fiction”.^43^

To counter the idea that sexual scientific knowledge could corrupt or seduce vulnerable people into sexual behaviours, sexual scientists often relied on the argument that nature was unchanging and could not be altered by external stimuli. In early twentieth-century Germany, for instance, when defending cases of improper publication, “homosexual emancipationists” rejected the seduction thesis by drawing on the idea that nature was fixed.^44^ As a result, the articulation of natural truths had no “wizardly” power to influence or shape homosexual desires. ^45^ If, for example, homosexuality was a biological, in-born or hereditary condition, then it could not be acquired and could not be promoted by books, magazines, films or teaching. Swiss psychiatrist Auguste Forel made the same point in a chapter on sex and pedagogy, stressing that the parts of the “sexual appetite, sensations and sentiments” that are “phylogenetic or hereditary” can “in no way” be changed by any form of pedagogy or sexual learning; they are “predetermined, and constitute the soil to be cultivated by education”.^46^ Yet, as we shall see below, this notion of nature as fixed and unchanging sat alongside another understanding of the natural sexual instinct as responsive to external stimuli within sexual scientific debates.

## Sex Researchers as Liberators of Nature

In addition to justifying sexual scientific work on the grounds that the study of nature could not in itself be inappropriate or damaging, sex researchers had an even more powerful response to the threat of censorship: they argued that it was the *absence* of sexological knowledge that was harmful and corrupting. This strategy sought to turn the debate about censorship on its head in arguing that open and objective knowledge about sex was natural and beneficial whereas silence and repression were damaging and unnatural. In this regard, sex researchers sometimes presented themselves as the heroic liberators of both sexual knowledge and sexual nature. Their alleged heroism lay precisely in their daring challenge of the repressive forces of censorship and silence. Sex researchers authorized their work by claiming to offer a more truthful and impartial assessment of sexual questions that could replace biased, moralistic and dogmatic understandings of sexuality held in the past. They became the defenders and protectors of what they constructed as the natural truth of sexuality. During the Bedborough trial of 1898, for instance, a defense committee consisting of high-profile progressives presented Ellis in this way. One of them, Robert Buchanan, characterized *Sexual Inversion* as “a noble bit of work, done in the interests of suffering humanity”. Using colonialist distinctions between the civilized and primitive, the ban was presented as an offence to modern society: it was an “insult to a man of science […] more worthy of savages [sic] than of sane men living in the nineteenth century”.^47^

Sex researchers themselves saw their work as a necessary and valuable exposure of the natural truth of sexuality. The introductions to many sexological texts robustly asserted their value and importance in a world dominated by sexual repression and ignorance. The introduction to Carpenter’s *Intermediate Sex* (1908) presents the subject as being of “great” and “growing importance” and part of a broad and substantial literature on the topic in “scientific works, medical treatises, literary essays, romances, historical novels, [and] poetry”, and much needed by readers who are also imagined as a wide (albeit educated and professional) constituency of “medical men, teachers, parents, magistrates, judges and the like”.^48^ English palaeobotanist and birth control advocate Marie Stopes presented her best-selling *Married Love* (1918) as “knowledge” for the “service of humanity” that could replace “years of heartache and blind questioning in the dark”.^49^ Examining sex was constructed as a central scientific duty; sexology – across the nineteenth and twentieth century, in all its variation – was authorized fulfilled the modern scientists’ responsibility to strip away social taboos, conventions and, in particular, to fight the determined concealment of sexual knowledge.

Some sex researchers pushed this argument further by claiming that it was those who sought to prevent sex research and its dissemination that damaged society. They produced a catalogue of social ills that would be solved by the sexual scientific knowledge. When radical free love journal *The Adult* reported on the *Sexual Inversion* trial, ^50^ in which some of its own articles were also indicted, it was sexual ignorance that was presented as the blight on British society. It was the lack of knowledge that caused, among other things, public schools to be a hot bed of unnatural vice. By contrast, the solution, indeed the very progress of civilization, required the dissemination of sexual knowledge.^51^

In some cases, the “sexual perversions” studied by sex researchers were even said to be caused by the refusal to treat sex as a natural part of human knowledge. Féré maintained that sexual perversions arose when false facts about “sexual physiology and pathology” went unchecked without explaining their “real significance”.^52^ For Féré, this was counterintuitively true in the case of biologically caused perversions, where a powerful, irresistible, biological instinct might be driven into an abnormal direction if the significance of facts relating to sexual physiology and pathology are not pointed out. ^53^ Medical doctor Bernard S. Talmey’s 1919 textbook on sexual attraction also saw the withholding of information about the natural facts of sex and reproduction as the cause of sexual depravity: “it is surprising that until recently sexuality was not looked upon with great favour, and that a sane knowledge of sex and reproduction was assiduously withheld from the people. […] But to the really innocent and pure all things are pure […] [A] false sense of shame […] [has created a] diseased imagination, depraved beyond all hope”.^54^ This was a barbed argument which in appropriating the idea of innocence and ignorance as a form of purity portrays those seeking to hide sexual knowledge as suffering from a depraved imagination.

The politics of censorship thus became a particular focus of sexual scientific research which sex researchers confronted directly. Rather than simply defending their research or justifying their publication and dissemination strategies, they wrote directly about the ill-effects of censorship. By the time of the World League for Sexual Reform Congress in 1929, which was held in London, an entire strand of the program was devoted to the discussion of censorship, which included H.F. Robenstein on “Sex, Censorship and Common Sense in England”, Laurence Housman on “Sex and Censorship”, John van Druten on “Sex and Censorship in the Theatre”, Ivor Montagu on “The Censorship of Sex in Films”, Marie Stopes on “The Scientific Consideration of Population Problems and Interference with the Freedom of Population”, and George Ives on “The Taboo Attitude”.^55^

This approach, which attacked the attempt to restrict sexual knowledge as dangerous and a root cause of society’s sexual ills, was part of a broader historical critique of modern society embraced by many sex researchers. By the beginning of the twentieth century, the characterization of the nineteenth-century world as repressed had become a common trope of progressive opinion. Often labelled as “Victorian”, this theme was not limited to authors writing in an English context, although their voices were especially well developed and strong. What it meant to be modern in the early twentieth century became increasingly defined by a contrasting view of sexual knowledge. For example, the establishment of the BSSSP in 1913-1914 saw the damage done by sexual censorship in the past as core to its mission and called for members to assist in undoing the damage done when moral judgment impedes sexual knowledge.^56^

The idea that censorship was a product of the harms wrought by a Judeo-Christian puritanism that had been strengthening its grip on western Europe since the medieval period was a favourite theme among many writers. Austrian psychiatrist Richard von Krafft-Ebing affirmed that sexual science’s mission was to do away with the “ridiculous prudery [lächerlichen Prüderie]” of the past.^57^ In 1901, sex researcher J.A. Godfrey sought to demonstrate the damages done to women’s natural sexual potential by historically inherited puritanical views:

thanks to our puritanical view of sex matters in general, the woman may grow up with the idea that sexual manifestations are in themselves degrading. […] [E]very sexual impulse is restrained at its beginning, every thought of love opposed as an unpardonable weakness. After years of such a process […] she will have ceased to respond [sexually]. […] She has become an abnormality, a being sterile alike in body and heart.^58^

When Magnus Hirschfeld gave the opening address at the World League for Sexual Reform in 1929, he reflected on the achievements of sexual science since the publication of his early treatise *Sappho and Socrates* in 1896. Proclaiming science as the “mouthpiece of nature”, he championed sexology as a new way of life that had replaced the harmful silence of Christianity with a pure and sacred truth: “[In 1896], ignorance was synonymous with innocence, and silence on all sexual subjects was regarded as sacred. Many changes have taken place since then and to-day we realise that in sexual matters ignorance is not innocence but guilt, and that it is our sacred duty to break through the conspiracy of silence.”^59^

This critique of the West as governed by an out-of-date Christian prudery was further bolstered by a romanticized and colonialist construction of many non-Western cultures, especially South Asian cultures, as practicing an open approach to sexual matters that allegedly resulted in greater enlightenment and improved health.^60^ The idea that ancient Sanskrit (and other Asian) erotic texts provided healthy instructions in sexual knowledge, for instance, had become a common theme by the early twentieth century.^61^ Indeed, some scholars saw this knowledge as a form of ancient sexology that modern European sex researchers were re-inventing. From the end of the nineteenth century onwards, orientalists saw the translation of such texts as valuable in these precise terms; they allegedly provided Western audiences with a form of educative sexual science comparable to that of modern sexology. For instance, the alleged translation of an Indian text for an English-speaking audience, *Rati Sastram*, was subtitled: *Hindu System of Sexual Science*. First published in 1898, the 3^rd^ edition in 1904 uses its back pages to advertise another work, *The Dictionery* [sic] *of Sexual Science or The English Translations of Hindu Sexual Science*, which promises “full and detailed information regarding the sexual science of new life”.^62^ Similarly, Carpenter used the apparent openness of Sanskrit erotic texts to charge modern censors with being the ones with impure thoughts and corrupted minds. When we refuse to translate Sanskrit texts, he argues, we show our own degradation from the “pure and pious sentiment” of the ancient authors: “Our public opinion, our literature, our customs, our laws are saturated with the notion of the uncleanness of Sex”, such that until this “dirty and dismal sentiment with regard to the human body is removed there can be little hope of anything like a free and gracious public life”.^63^

Seeing the censorship of sexual science and the dissemination of sexual knowledge as damaging and productive of immorality was asserted with particular force from the 1910s onwards, when the argument that sexual knowledge was an essential part of the education of youth was more frequently articulated. Carpenter, however, had already made this central to his articulations of the value of sexual science a decade earlier. Carpenter’s account of the Bedborough trial argued that educationalists had the most to benefit from the publication of decent and scientific works, since schools were already corrupted and impure places. The correct response was not censorship, or the preservation of youthful ignorance, but the instruction of all schoolmasters in sexual matters.^64^ In 1896, he justified the publication of his own series of pamphlets entitled *Love’s Coming of Age* on the grounds that publication will “reverse the corruption of children” brought by ignorance: “That we should leave our children to pick up their information about the most sacred, the most profound and vital, of all human functions from the mere gutter […] seems almost incredible”.^65^ When corresponding with the British Museum about the private case, Carpenter drew attention to the need of young people and their teachers for this information: “the present ignorance on the subject is a serious drawback in a vast number of cases, both to young folk themselves and to their elders whose business it may be to act as their guides”.^66^ Indeed, Carpenter sent copies of his books to prominent (boys’) schools.^67^

By the 1920s, sex researchers contributed to a growing literature of sex advice aimed at young people or their educators. Confronting the censorship of sexual material and tackling the problems of sexual ignorance became the explicit publishing purpose of a whole range of sex education texts and marital advice guides. The English art historian and sex educator Catherine Hartley’s *Sex Education and National Health* (1920) includes a preface from a teacher converted to the view that a healthy approach to sex is an informed one: “As a public School [sic] master […] I began by treating rows about sexual vice as something too horrible to speak about […] [, but I now believe that we] need co-education, married teachers and books like this”. It was important to stop seeing “ignorance as a fragile possession to be protected” and instead challenge the “conspiracy of silence”.^68^ Similarly, American social hygienist Maurice A. Bigelow’s sex education lectures delivered at Columbia University in 1916 claimed that “the policy of silence has been a gigantic failure, because it has not preserved purity and innocence and because it has allowed grave evils, both hygienic and moral, to develop under the cloak of secrecy. […] But why should we expect the human race to make progress when sexual problems have been kept in darkness?”^69^

## The Uses of Self-Censorship

In drawing attention to the damages wrought by what many saw as a repressive sexual culture that fostered silence and ignorance, sex researchers simultaneously acknowledged that external influences could corrupt natural impulses. As argued above, this idea was underpinned by conceptualisations of the sexual instinct as responsive to external stimuli. This created a dilemma for sexual science: constructing the sexual instinct as volatile and open to influence made it difficult to deny that there were circumstances in which sexological knowledge itself had to be regulated and controlled. Indeed, sex researchers frequently acted as censors when publishing and circulating their own and other people’s works, thus accepting and reinforcing anxieties about the damaging potential of sexological knowledge.

When working with these sources, it can be difficult to discern the intentions and motivations of sex researchers. It is clear that authors, translators, editors and publishers were navigating a publishing world and legal environment in which some concessions were necessary to ensure that their work could be disseminated.^70^ In this sense, accepting or even initiating forms of censorship was a practical way of managing external pressures. At the same time, and more fundamentally, the very idea that sexual science had a vital place in modern society hinged on the argument that the sexual instinct was not simply a natural force that would automatically unfold in ways that would serve the health of the individual, the progress of society and the development of the species. Instead, presenting the sexual instinct as a volatile element of human nature that required external guidance was key to legitimating sexual scientific work.

Sexological publications were frequently issued with frontispieces or prefaces clarifying that the intended audience was limited to certain groups and their print runs were often small. As we have already seen in relation to the English edition of Bloch’s work above, inserting these statements into prefaces was often seen as sufficient to ensure that a book could be published. Chief amongst the intended readership were scientists and medical doctors as well as jurists, but it also frequently included teachers and other professionals involved in sex education. Even the texts that envisaged the widest readership frequently specified that readers should be adults (and often married). Ellis argued that *Sexual Inversion* did not seek a general readership and was therefore scientific and serious.^71^ Sometimes the list of intended readers were very long; for instance, Austrian psychiatrist Richard von Krafft-Ebing introduced German physician Albert Moll’s *Die Konträre Sexualempfindung* by saying the book was aimed at the professional man [“Fachmann”] and doctors, police officials, judges, lawyers, historians, psychologists, anthropologists, sociologists, educators of youth and of society, and policy makers.^72^ The same caution was applied to conferences and congresses. When English animal geneticist F.A.E. Crew invited Finnish anthropologist Edvard Westermarck to serve on the organizing committee of the second Congress of the International Society for Sex Research in 1929, he stressed that “both the Society and the Congress are solely interested in the purely scientific aspects of the biology of sex. They are in no way and at no time interested in any kind of propaganda. The Congress will not be open to the public and membership and admission will be restricted to serious students in this field of science.”^73^

As with other forms of censorship, it is highly doubtful that these actions served to regulate the reach of sexological knowledge reliably or systematically.^74^ Statements aiming to limit the audience of sexological findings fulfilled other purposes. They offered a declaration of intent to appease publishers, deter prosecuting authorities and to alleviate potential anxieties on behalf of professional participants who did not label themselves as sex researchers or sexual scientist. The repeated assertions on behalf of sex researchers that access ought to be limited also reinforced the idea that sexological findings might have a harmful impact if they fell into the hands of the “wrong” readers. While this argument could clearly be used against the dissemination of sexual scientific findings, it also presented sexual science as a powerful and serious form of knowledge that professional readers needed to engage with to be able to guide and control the development of the sexual instinct.

Sex researchers used different methods to limit access to sexological knowledge themselves, including self-censorship. The decision to publish parts of sexological studies in Latin, for example, Krafft-Ebing’s *Psychopathia Sexualis* or Talmey’s *Love: A Treatise on the Science of Sex-Attraction*, is one of the most widely noted examples of sexological self-censorship.^75^ But there were also more subtle ways of controlling language: Krafft-Ebing, when introducing Albert Moll’s *Die Kontraere Sexualempfindung* in 1891, for instance, wrote it would be desirable – in principle – for a very wide audience to read the book. However, this would not be possible because of Moll’s stylistic and terminological choices: “Certainly, it [the book] cannot be treated as popular reading, and the author has ensured this won’t be case through stylistic choices and by drawing on learned language; but every academically trained reader will be able to understand the book.”^76^ Other sources reveal moments of self-censorship as texts were prepared for publication. For instance, before publishing medical doctor F.B. Rockstro’s *A Plain Talk on Sex Difficulties*, the lawyer Gerald Gardiner was asked to comment whether the book was likely to be censored. In Gardiner’s opinion, “prosecution [was] highly improbable”, but if the book were to be prosecuted for any reason, he could not guarantee that it would not be banned.^77^ He therefore suggested that Rockstro alter the “colloquial” language^78^ and reduce his own commentary “upon the desirability of changing the basis of our sexual morality”. This demonstrates that sex researchers paid careful attention to their use of language and employed it strategically to try and control who had access to sexological knowledge.

In addition, sexological networks were themselves responsible for policing the circulation of sexological literature. For instance, the BSSSP – while generally seeking to make sexological publications more accessible to wider audiences – refused to share their own publications with non-members if texts dealt with more ‘controversial’ topics. In 1927, a non-member called Dr M.B. Arnold ordered *Some Friends of Walt Whitman* and *The Social Problem of Sexual Inversion* and was only sent the Whitman publication. The reply from the BSSSP suggests that Arnold had to undergo an interview before he was able to receive the second publication. Becoming a member was not an option for everyone either. For instance, with regard to age, members had to be 25 or above in the early years of the society.^79^ While their membership protocols were often relaxed,^80^ the age limit was occasionally imposed; in 1923, for example, they turned down a female applicant who was under the age of 25.^81^ This demonstrates that those involved in publishing scientific sex research made active choices to restrict the access to their findings. While this was certainly done in a genuine bid to protect themselves against censorship or to protect professional reputations, it nevertheless meant that sex researchers themselves reinforced the idea that (at least some) readers had to be protected from sexological knowledge.

Unsurprisingly, as several of the examples discussed so far already indicate, the corruption of younger minds was at the very forefront of these concerns. In addition to stressing the need for readers to be professional and educated, they also had to be mature. At times, sexological publications were explicit in saying that sexological knowledge must not fall into the hands of young readers. As noted above, the English edition of Bloch’s *The Sexual Life of Our Times* was published with a preface stating that the book was aimed at a broad professional public, but should certainly be classified as “adult literature”.^82^ Reviews of sexological publications reinforced this point: an anonymous review of *Sexual Inversion* states that it would be most “undesirable” for the book to be read by “immature and half-educated people”.^83^ In the early 1930s, Rockstro’s *A Plain Talk on Sex Difficulties* (1934) – a best-selling title published by the BSSSP – was advertised with flyers stating it was only for married adults.

This emphasis on maturity created a tension between the need to both inform *and* protect young people from sexual knowledge, which became central to the self-policing and self-authorisation of sexual science. Ellis’s *Little Essays of Love and Virtue* (1921) was explicitly aimed at young people and written in accessible language. In his preface, Ellis presented the book as a young readers’ edition of the “fundamental principles” previously set out or implied in his *Studies in the Psychology of Sex*.^84^ The book covered discussions of masturbation, heterosexual sex within marriage, female sexuality, reproduction and eugenics, but it did not discuss homosexuality, sadomasochism and other ‘perverse’ sexualities discussed at length in his *Studies*. This indicates that sex researchers were selective in choosing which aspects of sexual knowledge they should communicate to younger audiences and in what ways.

This reticence was not just due to fears about censorship, but also because of genuine concerns about the impact that sexual knowledge could have on receptive readers. As noted above, some sex researchers relied on the idea of nature being inborn, fixed and unchangeable to defend themselves and their work against accusations of seductions. However, despite the simplicity of this defence, the theories of sexuality that sexual science developed were not, in fact, so straightforward. The seduction thesis was never comprehensively rejected, and certainly not in favour of an exclusively inborn or fixed model of the sexual instinct.^85^ Very few works of sexual science saw all expressions of sexuality as exclusively fixed or inborn. Theories of sexuality recognized and explored variety and change in ways that made it impossible to sustain an anti-censorship argument on the grounds of a simple, universal in-born model. It was recognized by most sex researchers that biological predispositions co-existed with environmental, habitual or climactic factors. A model which saw the sexual instinct as variable, adaptive and responsive to external stimuli ensured that change was inherent and built into models of human sexuality.

As a result, sexual science was not immune to widespread anxieties that modernity, urbanisation, new technologies and media like “cinema shows” could alter individuals’ allegedly natural heterosexual desires.^86^ According to Stopes, this could lead to a lack of heterosexual intimacy and diminish women’s “spontaneous sex-impulse” altogether.^87^ Although often invested in congenital readings of homosexuality, Ellis also suggested that “there are many influences in our civilisation today which encourage” expressions of homosexual desire.^88^ German physician Albert Moll was even more explicit in identifying the impossibility of using biological predisposition to underpin arguments against censorship. He pointed out that, regardless of biological predisposition, it was entirely possible for literature to redirect fantasies and desires: in cases when individuals “choose reading material that leads their fantasies in a homosexual direction, the normal sexual life is increasingly suppressed and the homosexual element is strengthened”. For Moll, literature with homosexual content could corrupt a heterosexually constituted person away from normal sexuality and towards homosexuality.^89^ Similarly, while Forel argued that sexual information could not alter people’s fundamental impulses or desires, which were generally hereditary and fixed, he nevertheless maintained that the sexual instinct could be subtly calibrated or disarranged as a result of external influence. A hereditary tendency towards homosexuality, for example, could be increased as a result of “seductive influence” or it could be decreased via the experience of heterosexual love.^90^ For Forel, then, education was essential to ensure that hereditary impulses were directed and shaped via appropriate external influences. To achieve this, individuals needed to be provided with “instruction on the relations of the sexes, in due time and in a serious manner, instead of replying to ingenuous questions by pious falsehood, by equivocation, or by an air of mystery”.^91^

Questions about which kind of sexological knowledge should be made accessible to certain individuals at specific moments in their development drove sexual scientific debate in the early twentieth century, especially as the question of delivering formal sex education became increasingly debated. For instance, psychoanalyst Barbara Low, a member of the BSSSP, stated in her lecture to the 1929 London Congress of the World League for Sexual Reform that sex education for children was crucial, but the question of how it should be delivered required careful study and deliberation. She stressed that “we have not solved the problem of which persons are best suited to give such instructions [to the young], nor under what conditions nor at what stage of development it shall be conveyed.”^92^

Instead of simply opposing censorship, then, sex researchers often conceded that it was necessary to manage and control information about sex responsibly. The needs of specific audiences had to be considered and material had to be carefully curated for each specific cohort. Far from having a straightforwardly repressive impact on the development of sexual science, these contestations led to vibrant debate and exchange, which connected sex researchers with differing political and intellectual investments. More than that, the underpinning suggestion that the sexual instinct was open to external stimuli, including sexual scientific knowledge, meant that sexual science could legitimize itself as a field whose contributions were crucial to the management of the sexual instinct and the development of civilization and society as a whole.

## Conclusion

This article has demonstrated that the reality and threat of censorship played a constitutive, and often misunderstood, role in the emergence of British and German sexology. Debates about censorship and obscenity were fueled by anxieties about the influence of sexual knowledge on different audiences. Although often opposed to specific censorship decisions, sexual scientists fundamentally agreed that the sexual instinct was changeable, open to external stimuli and part of evolutionary processes of development. For this reason, sex researchers argued that sexual knowledge was both essential to the health (of the individual and society) and a potentially dangerous influence that needed to be controlled carefully. When responding to the possibility that their work might be censored, sex researchers took a position that was informed by these insights. On the one hand, sex researchers criticised contemporary European censorship regimes as repressive and damaging. In particular, they saw the regulation of sexual knowledge as the restriction of natural truths about human sexuality. On the other hand, they conceded that their work could have a potentially damaging influence in some contexts. The model of the sexual instinct that they developed constructed sexual nature as a volatile force that needed careful management, including via the regulation of sexual information. This posed a challenge to the publication and circulation of works of sexual science. In often paradoxical ways, sex researchers appealed to the need both to protect and liberate sexual nature from external corruption. Insisting that sexual nature could be damaged through external influences allowed sex researchers to cast themselves in the role of heroic liberators in disseminating scientifically grounded information, but it also meant acknowledging that sexual knowledge could be corruptive if (mis)read by the “wrong” audience. As a result, sex researchers were not simply victims of censorship, but often played an active role in censoring and regulating the production and circulation of their own findings.

## Footnotes

3 Chris Nottingham, *The Pursuit of Serenity: Havelock Ellis and the New Politics* (Amsterdam: Amsterdam University Press, 1999), 209-10.

4 Robert Beachy, *Gay Berlin: Birthplace of a Modern Identity* (New York: Vintage Books, 2015), 166-69; Ina Linge, ‘Sexology, Popular Science and Queer History in *Anders Als Die Andern (Different from the Others)’, Gender & History* 30, no. 3 (October 2018), 595–610.

5 Heike Bauer, *The Hirschfeld Archives: Violence, Death, and Modern Queer Culture* (Philadelphia: Temple University Press, 2017), 78–101.

6 On sexology as a loosely defined field, see Kirsten Leng, *Sexual Politics and Feminist Science: Women Sexologists in Germany, 1900-1933* (Ithaca: Cornell University Press, 2018), 14-34.

7 Following Potter, we understand censorship as a process that involves multiple actors across a broad network of co-constitutive systems of regulation and control, including self-censorship and editorial censorship as well as censorship as exerted by libraries and places of education and government officials and the law. See Potter, *Obscene Modernism*, 14-41; Robert Spoo, *Modernism and Law* (London: Bloomsbury, 2018), 45-78.

8 Michel Foucault, trans. Robert Hurley, *The History of Sexuality Vol. 1* (Harmondsworth: Penguin, 1990), 15–35. See also Annette Kuhn, *Cinema, Censorship and Sexuality, 1909-1925* (London: Routledge, 1989); Helen Freshwater, *Censorship & Cultural Regulation in the Modern Age* (Amsterdam and New York: Rodophi, 2004), pp. 217-37.

9 Important scholarship in modernist studies has explored the generative influence of censorship on literary production in the late nineteenth- and early twentieth century. See, for instance, Celia Marshik, *British Modernism and Censorship* (Cambridge: Cambridge University Press, 2006); Rachel Potter, *Obscene Modernism: Literary Censorship and Experiment 1900-1940* (Oxford: Oxford University Press, 2013); Paul Vanderham, *James Joyce and Censorship: The Trials of Ulysses* (Basingstoke: Palgrave, 1998). Censorship regimes, including attempts to curtail the circulation of obscene materials, affected both scientific and literary writers. Although a closer investigation lies beyond the scope of this article, the resulting connections are one of the reasons why literary modernism and sexual science need to be understood as intersecting fields of knowledge production and expression.

10 Although we demonstrate how the concept of “nature” was mobilised in conflicting ways, our article does not seek to do justice to the complex and contradictory ways in which nature and the natural were constructed in sexual science.

11 On the history of censorship and sexual knowledge, see Walter M. Kendrick, *The Secret Museum: Pornography in Modern Culture* (Berkeley: University of California Press, 1996); H. G. Cocks, “Saucy Stories: Pornography, Sexology and the Marketing of Sexual Knowledge in Britain, c. 1918-70”, *Social History* 29, no. 4 (2004): 465-84; Sarah Bull, “A Purveyor of Garbage? Charles Carrington and the Marketing of Sexual Science in Late-Victorian Britain,” *Victorian Review* 38, no. 1 (2012), 55-76; H. G. Cocks, “Reading Obscene Texts and their Histories,” *Media History*, 18, no. 3-4 (2012), 275-88.

12 French authorities, for example, tended not to prosecute works of sexual-scientific thought (as opposed to mass-produced sexually explicit material) in part because an 1882 law on “immoral books” meant that only a jury in the highest criminal court could consider a case. See Sarah Bull, “More than a Case of Mistaken Identity: Adult Entertainment and the Making of Early Sexology,” *History of the Human Sciences* 34, no. 1 (2021): 12.

13 Sarah L. Leonard, *Fragile Minds and Vulnerable Souls: The Matter of Obscenity in Nineteenth-Century Germany* (Philadelphia: University of Pennsylvania Press, 2015), 21; 26.

14 Leonard, *Fragile Minds*, 193.

15 As Sarah L. Leonard argues in relation to unified Germany, “[r]egulating the quality and effects of sexual arousal and protecting sexual honor and modesty became the central principles at stake in these laws”. See Leonard, *Fragile Minds*, 193. With regard to Britain, see Katherine Mullin, “Poison More Deadly than Prussic Acid: Defining Obscenity after the 1857 Obscene Publications Act (1850–1885),” in *Prudes on the Prowl*, ed. David Bradshaw and Rachel Potter (Oxford University Press, 2013), 11–29; Philip Kuhn, “The Sexual Life of Our Time: Medical Censorship in Early-20th-Century England,” *History of Psychology* 23, no. 1 (Feb 2020): 40-61.

16 For more on degenerationist thinking in relation to obscene materials, see Kathleen Frederickson, *The Ploy of Instinct: Victorian Sciences of Nature and Sexuality in Liberal Governance* (New York: Fordham University Press, 2014), 46.

17 Annie Besant’s argument that “medical or physiological” materials do not elicit sexual responses in readers did not help her case during the 1877 censorship trial. See Frederickson, *Ploy of Instinct*, 45.

18 William L Langer, “The Origins of the Birth Control Movement in England in the Early Nineteenth Century”, *The Journal of Interdisciplinary History* II (Spring 1975): 685.

19 Nottingham, *The Pursuit of Serenity*, 209-10.

20 A newspaper cutting on the case is attached to the Wellcome Library copy of Bloch’s *The Sexual Life of Our Time*. The original article appeared in “London Police Courts: Publishers’ Summoned,” *The Daily Telegraph*, no. 16755 (Jan 7^th^ 1909): 4. The radical press also reported on the case, arguing for the appropriateness of the book. See *The New Age: A Weekly Review of Politics, Literature and Art* 4. no. 12 (January 14^th^ 1909): 235.

21 F. Raymond Coulson and George Astor Singer, *Darwin on Trial at the Old Bailey* (London: University Press, 1900), 83.

22 Leonard discusses a 1882 legal opinion on obscenity legislation drafted by staff from the law faculty at the University of Leipzig. See Leonard, *Fragile Minds*, 200.

23 Bull, “More Than a Case”, 11.

24 Leonard, *Fragile Minds*, 190-91.

25 Robert Beachy, “The German Invention of Homosexuality,” *The Journal of Modern History* 82, no. 4 (December 2010): 821.

26 Laurie Marhoefer, *Sex and the Weimar Republic: German Homosexual Emancipation and the Rise of the Nazis* (Toronto: Toronto University Press, 2015), 42-9.

27 For more on the contrasts between Britain and the Continent, see Sean Brady, *Masculinity and Male Homosexuality in Britain, 1861-1913* (Basingstoke: Palgrave, 2009), 9. Brady argues that Britain was an exception within Europe, since British scientific networks were particularly opposed to the discussion of sexual matters and sexological writings subject to extensive censorship.

28 Havelock Ellis, *The New Spirit* (London: Constable & Company, 1926), 8.

29 Paolo Mantegazza, *The Sexual Relations of Mankind* (New York: Eugenics Publishing Company, 1935), vi.

30 F. Raymond Coulson and George Astor Singer, *Darwin on Trial at the Old Bailey* (London: University Press, 1900), 84.

31 Ferdinand Karsch-Haack quoted in Ralph Matthew Leck, *Vita Sexualis: Karl Ulrichs and the Origins of Sexual Science* (Urbana: University of Illinois Press, 2016), 75.

32 M.E. Robinson, “The Sex Problem,” *International Journal of Ethics* 21 (1911): 332.

33 Coulson and Singer, *Darwin on Trial*, 23.

34 Coulson and Singer, *Darwin on Trial*, 23.

35 Coulson and Singer, *Darwin on Trial*, 21.

36 Geoffrey Mortimer’s article “Reticence and Prurience”, which comments on the Bedborough Trial, is reprinted in Coulson and Singer, *Darwin on Trial*, 106-7.

37 Magnus Hirschfeld, *Naturgesetze Der Liebe: Eine Gemeinverständliche Untersuchung Über Den Liebeseindruck, Liebesdrang Und Liebesausdruck* (Berlin: Alfred Pulvermacher & Co., 2012), n. pag.

38 For more on the BSSSP, see Laura Doan, “Troubling Popularisation: On the Gendered Circuits of a ‘Scientific’ Knowledge of Sex,” *Gender & History* 31, no. 2 (2019): 304–18; Lesley A. Hall, “’Disinterested Enthusiasm for Sexual Misconduct’: The British Society for the Study of Sex Psychology, 1913-47,” *Journal of Contemporary History* 30, no. 4 (1995): 665–86.

39 Many of Britain’s academic libraries collected and preserved sexological publications and established access regimes to allow some readers to use them. For instance, students wishing to access sexological materials in the Phi Collections at Oxford’s Bodleian Library and the Arcana Collections at Cambridge’s University Library needed to produce a written endorsement from a tutor. See Lloyd (Meadhbh) Houston, “Towards a History of the Phi Collection, 1882-1945,” *Bodleian Library Record*, 28.2 (October, 2015), 179-94; Liam Sims, “‘Scandalous and Libellous Books’: the Arc Collection at Cambridge University Library”, *Transactions of the Cambridge Bibliographical Society* XV/4 (2015), 625-645. Similar arrangements existed in many other European museums. See Catherine Johnson-Roehr, “In Search of Secret Museums,” *The Journal of Sex Research*, 48, no. 1 (2011), 94-95.

40 Edward Carpenter Correspondence with the British Museum, 27^th^ June to 4^th^ July 1912, MSS 184-4, Carpenter Collection, Sheffield Archives.

41 Examples include Marie Stopes’ best-selling publications *Married Love* (1918) and *Wise Parenthood* (1919) as well as Havelock Ellis’s *Little Essays of Love and Virtue* (1921). Stopes’s work was also adapted for the screen as *Maisie’s Marriage* (1923). Hirschfeld’s films included *Anders als die Andern* (1919) and *Gesetze der Liebe* (1927).

42 For more on the relationship between literature and sexology, see, for instance, Heike Bauer, *English Literary Sexology: Translations of Inversion, 1860-1930* (Basingstoke: Palgrave, 2009); Jana Funke, *Sexological Modernism: Queer Feminism and Sexual Science* (Edinburgh: Edinburgh University Press, 2023); Anna Katharina Schaffner, *Modernism and Perversion: Sexual Deviance in Sexology and Literature, 1850-1930* (Basingstoke, Palgrave, 2011).

43 James Douglas, “A Book That Must Be Suppressed”, reprinted in *Palatable Poison: Critical Perspectives on The Well of Loneliness*, ed. Laura Doan and Jay Prosser (New York: Columbia University Press, 2001), 36-38.

44 Laurie Marhoefer, “‘The Book Was a Revelation, I Recognized Myself in It’: Lesbian Sexuality, Censorship, and the Queer Press in Weimar-Era Germany,” *Journal of Women’s History* 27, no. 2 (2015): 63.

45 Marhoefer, “Lesbian Sexuality,” 63.

46 Auguste Forel, *The Sexual Question: A Study of the Sexual Life in All Its Aspects: Physiology, Psychology, Sociology* (London: Rebman, 1908), 470.

47 Reported in *The Sketch*, November 2 1898, see cutting in Ives Scrapbook, Beinecke Rare Book and Manuscript Library, Yale U, GEN MSS 426, Volume 4, 1903, 3.

48 Edward Carpenter, *The Intermediate Sex: A Study of Some Transitional Types of Men and Women* (London: George Allen & Unwin, 1921), 9-10.

49 Marie Carmichael Stopes, *Married Love* (London: G.P. Putnam & Sons, 1918), xiii.

50 Henry Seymour, “The Literary ‘Hall-Mark’ of the Old Bailey,” *The Adult*, 2, no. 11 (1898): 323-325.

51 For more on *The Adult*, see Sarah L. Jones “‘As Though Miles of Ocean Did Not Separate Us’: Print and the Construction of a Transatlantic Free Love Community at the Fin de Siècle,” *Journal of Victorian Culture* 25, no. 1 (2020): 95–109.

52 Charles Féré, *The Evolution and Dissolution of the Sexual Instinct* (Paris: Charles Carrington, 1904), xxv.

53 Féré, *Evolution and Dissolution*, xxv.

54 Bernard S. Talmey, *Love, Treatise on the Science of Sex-Attraction: For the Use of Physicians and Students of Medical Jurisprudence* (New York: Practitioners’ Publishing Company, 1919), 9.

55 Ivan Crozier, “‘All the World’s a Stage’: Dora Russell, Norman Haire, and the 1929 London World League for Sexual Reform Congress,” *Journal of the History of Sexuality* 12, no 1 (2003): 31.

56 BSSSP, *Policy & Principles*.

57 Richard von Krafft-Ebing, “Vorwort zur Ersten Auflage,” in Albert Moll, *Die Konträre Sexualempfindung* (Berlin: Fischer’s Medicin Buchhandlung, 1899), III. All translations are the authors’ own.

58 John Allen Godfrey, *The Science of Sex. An Essay Towards the Practical Solution of the Sex Problem* (University Press: London, 1901), 87.

59 Hirschfeld, Magnus, “Presidential Address,” in *World League for Sexual Reform: Proceedings of the Third Congress*, ed. Norman Haire (London: Kegan Paul, 1930), xi-xv.

60 On this, see Jana Funke, “Navigating the Past: Sexuality, Race and the Uses of the Primitive in Magnus Hirschfeld’s Travel Writings,” in *Sex, Knowledge & Receptions of the Past*, ed. Kate Fisher and Rebecca Langlands (Oxford: Oxford University Press, 2015), 111–34.

61 Far from constituting a Western scientia sexualis in opposition to an ars erotica, sexology produced romanticised readings of non-Western sexual texts and customs. It was these very interpretations of Eastern sexual cultures that were subsequently read by Foucault and shaped his *History of Sexuality*. See, Leon Antonio Rocha, “Scientia Sexualis Versus Ars Erotica: Foucault, Van Gulik, Needham,” *Studies in History and Philosophy of Science Part C: Studies in History and Philosophy of Biological and Biomedical Sciences* 42, no. 3 (2011): 328-43.

62 Abinash Chundra Ghose, *Rati Sastram, or the Hindu System of Sexual Science* (Calcutta: S.C. Seal, 1904). See also Durba Mitra, *Indian Sex Life: Sexuality and the Colonial Origins of Modern Social Thought* (Princeton: Princeton University Press, 2020), 23-24.

63 Edward Carpenter, *Love’s Coming of Age: A Series of Papers on the Relations of the Sexes* (Manchester: Labour Press, 1896), 18. Hirschfeld’s travel writings offer similar examples, see Magnus Hirschfeld, *Women East and West* (London: William Heinemann, 1935).

64 Edward Carpenter, “The Bedborough Case: To the Editor of the Saturday Review”, Cutting in Box 1/74, Carpenter Collection, Sheffield Archives.

65 Carpenter, *Love’s Coming of Age*, 8-9.

66 Edward Carpenter, Draft letter to the *BMJ*, 1909, MSS 184-20, Carpenter Collection, Sheffield Archives.

67 See, for instance, Eton College to Edward Carpenter, 31^st^ Oct, 1914, acknowledging receipt of *The Intermediate Sex*, MSS 184-25, Carpenter Collection, Sheffield Archives.

68 Catherine Gasquoine Hartley, *Sex Education and National Health* (London: Leonard Parsons, 1920), 9-10.

69 Maurice Alpheus Bigelow, *Sex-Education: A Series of Lectures Concerning Knowledge of Sex in its Relation to Human Life* (Macmillan: New York, 1924, 12; 15.

70 Kuhn, “The Sexual Life of Our Time”.

71 Havelock Ellis, *A Note on the Bedborough Trial* (New York: Privately Printed, 1925 [1898]).

72 von Krafft-Ebing, ‘Vorwort’, p. III

73 F.A.E. Crew letter to Edvard Westermarck, 17^th^ October 1929, EW VI (Skap 9&11), Turku Archive.

74 Despite these instructions, booksellers did not necessarily limit sales to specific people, and the advertising of works as “sensitive” could also enhance their appeal. For instance, when Victor Robinson’s *Encyclopedia Sexualis* was published in 1936, a reviewer criticised the titillating preface as a crude attempt to suggest the text included pornography with a view to increasing the works sales and notoriety. See Kingsley Davis, “Review of Victor Robinson, ‘Encyclopaedia Sexualis“’, *American Sociological Review* 1, no 6 (1936): 995-997. Ellis also recognised the positive publicity provided by censorship, calling the Bedborough trial “an enormous advertisement spontaneously offered by the police” (Ellis, *A Note on the Bedborough Trial*, 10).

75 Bernard S. Talmey, *Love: A Treatise on the Science of Sex-Attraction for the Use of Physicians and Students of Medical Jurisprudence* (New York: Practitioners’ Publishing Company, 1919). At the same time, Talmey’s book includes a dictionary of Latin words and their English translation to facilitate the readers’ understanding.

76 Krafft-Ebing, “Vorwort”, v.

77 Gardiner argues that Radclyffe Hall’s novel *The Well of Loneliness* would not have been prosecuted in 1928 were it not for the infamous *Sunday Express* article by James Douglas that brought the book to the censor’s attention in the first place. See British Sexological Society [BSSSP] Papers, Third Party Works, Folder 18, Box 3, 18 October 1933, Harry Ransom Center.

78 Gardiner states, for instance, that the word “orgasm” might be better than the word “come”. He explains: “The plain and sexually ignorant man for whom I take the book to be intended may very well never have heard of ‘orgasm’. But where two plain phrases are equally well be used, I think that the last ‘vulgar’ should be used for preference.” See Gerald A. Gardiner, ‘Untitled Notes’, Third Party Works, Folder 18, Box 3, 18 October 1933, British Sexological Society [BSSSP] Papers, Harry Ransom Center.

79 The age of admission was later changed to 21.

80 Correspondence suggests, for instance, that the requirement that two existing members should recommend a new member was often waved.

81 Correspondence C. Marion Mitchell to BSSSP, Folder 8, Box 15, 14^th^ May 1923, British Sexological Society [BSSSP] Papers, Harry Ransom Center.

82 Bloch, *The Sexual Life*, v. The Wellcome Trust edition of the text has a cutting from a newspaper reporting on the police actions against Rebman publishers for allowing the book to be sold without first asking questions about the professional status of the customer. Deemed too respectable to be publicly prosecuted, the case was brought before a magistrate who concluded that, while he agreed that opinion had changed on the importance and relevance of sexual science, the book was still legally categorised as obscene and should not be sold to anyone who asked for it. The copies in the possession of the police were then destroyed. The newspaper cuttings suggests that the defendants would appeal.

83 H. S., “Review of ‘Studies in the Psychology of Sex. Vol. I., Sexual Inversion’ by Havelock Ellis and ‘An Unknown People’ by Edward Carpenter,” *International Journal of Ethics* 9, no. 2 (1899): 262. Paradoxically, in accepting that children should be protected from almost all sexual material, sexual scientists added grist to the argument that adults should be comprehensively informed. Knowledge in the hands of adults would protect children and prevent them from being corrupted by improper knowledge. Thus, the reviewer suggests, it is “necessary that every schoolmaster, every criminal lawyer, we had almost said every head of a family, should be acquainted with its phenomena [sexual inversion]. Were the subject better understood, mistakes would be avoided that have ruined thousands of lives”.

84 Havelock Ellis, *Little Essays of Love and Virtue* (New York: George H. Doran Company, 2021), v.

85 On non-congenital models of sexuality within sexual science, see Benjamin Kahan, *The Book of Minor Perverts: Sexology, Etiology, and the Emergences of Sexuality* (Chicago: The University of Chicago Press, 2019).

86 Stopes, *Married Love*, 12–3

87 Stopes, *Married Love*, 33.

88 Ellis and Symonds, *Sexual Inversion*, 177.

89 Quoted in Marhoefer, “Lesbian Sexuality”, 66. Krafft-Ebing also regarded literature as potential inducement to perversion. See Anna Katharina Schaffner, “Fiction as Evidence: On the Uses of Literature in Nineteenth-Century Sexological Discourse,” *Comparative Literature Studies* 48 (2011): 173-4.

90 Forel, *The Sexual Question*, 217-8.

91 Forel, *The Sexual Question*, 141

92 Barbara Low, “Sexual Education: Some Psychoanalytical Considerations,” in Haire, *World League for Sexual Reform London Congress Proceedings*, 368.

